# Comparison between surgical and non-surgical management of primary hyperparathyroidism during pregnancy: a systematic review

**DOI:** 10.1007/s12020-024-03930-0

**Published:** 2024-06-25

**Authors:** Shezifi Eli, Shlomo Gozlan Gal, Zaina Adnan

**Affiliations:** 1 Bar-Ilan University, The Azrieli Faculty of Medicine, Safed, Israel; 2 Laniado Hospital, Netanya, Israel; 3 Department of Physiology and Cell Biology, Ben-Gurion University of the Negev, Beer-Sheva, Israel; 4 Division of Endocrinology and Metabolism, Clalit Medical Health Care Services, Haifa and Western Galilee District, Zvulon Medical Center, Haifa, Israel

**Keywords:** Primary hyperparathyroidism, Pregnancy, hypercalcemia, PTH, Parathyroidectomy

## Abstract

**Purpose:**

The management of primary hyperparathyroidism (PHPT) during pregnancy may be surgical or conservative. This study compared adverse outcomes between surgical and non-surgical treatments. Additionally, the study investigated the correlation between serum calcium values and complication rates.

**Methods:**

A systematic review of retrospective studies, case series, and case reports. Biochemical parameters, interventions, and outcomes of each pregnancy were recorded. The study population comprised two groups: the non-surgical and surgical groups. Adverse outcomes were categorized as maternal, obstetric, or neonatal.

**Results:**

The surgical and non-surgical groups consisted of 163 and 185 patients, respectively. A positive correlation was observed between the mean maternal gestational calcium value and both maternal and obstetric complication. Neonatal complications were more prevalent in patients treated conservatively across all maternal calcium values (p < 0.001). No significant differences were observed in maternal outcomes and overall obstetric outcomes between the study groups, albeit a higher mean serum calcium value in the surgical group (12.3 mg/dL) compared with the non-surgical group (11.1 mg/dL).

**Conclusions:**

Given the significantly lower neonatal adverse outcomes in the surgical group compared to the non-surgical group, along with non-inferior maternal and obstetric outcomes in the surgical group, the overall data of this study suggest that parathyroidectomy is favorable to non-surgical management even in cases of mild hypercalcemia.

## Introduction

Primary hyperparathyroidism (PHPT) is a relatively rare condition to encounter in women of reproductive age, with an estimated rate of 7.7–50 cases per 100,000 women of this population [1, 2].

Pregnancy is characterized by physiological changes in calcium metabolism which must be considered: calcium absorption increases twofold by third trimester, driven by vitamin D and parathyroid hormone-related protein (PTHrP). The latter is synthesized during pregnancy from the amnion, as well as breasts to lesser extent, and other reproductive tissues [3]. PTHrP acts similarly to parathyroid hormone (PTH), promoting epithelial growth and tissue differentiation in the fetus and acting as the primary stimulus for the active placental calcium pump [3]. Total serum calcium values decrease during pregnancy due to the increased intravascular volume, calcium placental efflux, and increased urinary output. Finally, ionized calcium levels remain unchanged, and PTH falls to the low-normal range [4–7].

The etiology of gestational PHPT is most often a parathyroid adenoma, and less commonly parathyroid hyperplasia (5–10%) or parathyroid carcinoma (<1%) [8, 9]. PHPT may also occur as part of an inherited syndrome, and genetic counseling has been recommended for patients younger than 40 carrying the disease [10, 11].

PHPT may be managed conservatively or surgically. Conservative treatment is typically recommended for asymptomatic patients, or those with mild hypercalcemia, or as a bridge to surgery. It includes close follow-up, electrolyte, and hormonal monitoring, along with hydration alone in mild to moderate patients or additional pharmacological therapy in cases of refractory hypercalcemia [7, 12, 13]. The definitive treatment for this condition is parathyroidectomy, which can be performed via cervical exploration or minimally invasive parathyroidectomy (MIP). [9, 14] Reports of successful ablation of the gland are also documented [15–17].

Data regarding pregnancy complication rates from gestational PHPT are variable. Norman et al. reported a significant difference in pregnancy loss in gestational PHPT [18], and citations across the literature claim complications of PHPT during pregnancy may reach as high as 67% of mothers and 80% of offspring [19–21]. However, more recent large cohorts have reported no increased risk of obstetric complications [22, 23]. Although several studies have shown increased risk of complications in non-surgical treatment compared to surgical treatment, current guidelines suggest surgery only when the patient is either symptomatic or with hypercalcemia exceeding 1 mg/dL above the normal limits [7, 24]. Similarly, surgical safety is controversial. While some studies conclude that surgery during pregnancy causes more adverse events than surgery on non-pregnant patients [25, 26], others conclude that the risks of parathyroidectomy are minimal, hence surgery should not be delayed when necessary [27–30]. A recent Chinese expert consensus concluded surgery should be considered regardless of the stage of pregnancy when hypercalcemia appears hazardous to the patient or fetus [7]. In mind of the above controversies, the current study compared the complication rates between surgical and non-surgical managements, utilizing all the relevant published data available.

## Methods

### Study design

A literature search on PubMed, ScienceDirect (Elsevier), and Google Scholar was performed using the terms: “pregnancy” or “gestation”, “hyperparathyroidism”, “PHPT”, and “parathyroidectomy”. Database included published studies between 1980 and 2023. The study population comprised of women who experienced primary hyperparathyroidism (PHPT) during pregnancy and either underwent parathyroidectomy during pregnancy (surgical group) or were managed conservatively (non-surgical group). Recorded data included the patient’s age, biochemical values, obstetric and medical history, time of diagnosis, etiology, presenting symptoms, management, and complications, which were subsequently classified as maternal, obstetric, or neonatal. We excluded reports involving significant comorbidities, such as metastatic non-parathyroid cancer or any condition unrelated to PHPT severe enough to require management in the intensive care unit (ICU) during pregnancy, reports with missing data, and patients treated with alternative management not addressed in this article’s scope. We excluded cases with calcium values exceeding 15 mg/dl as these were deemed rare and complex, leading to higher complication rates and outcome bias. Additionally, we excluded records involving ectopic parathyroid adenomas, parathyroid carcinomas, and patients positive for multiple endocrine neoplasia type 1 (MEN1) as these rare cases were correlated with extended hospital stays, alternative management strategies, and complicated outcomes.

### Data collection

**Fig. 1:**
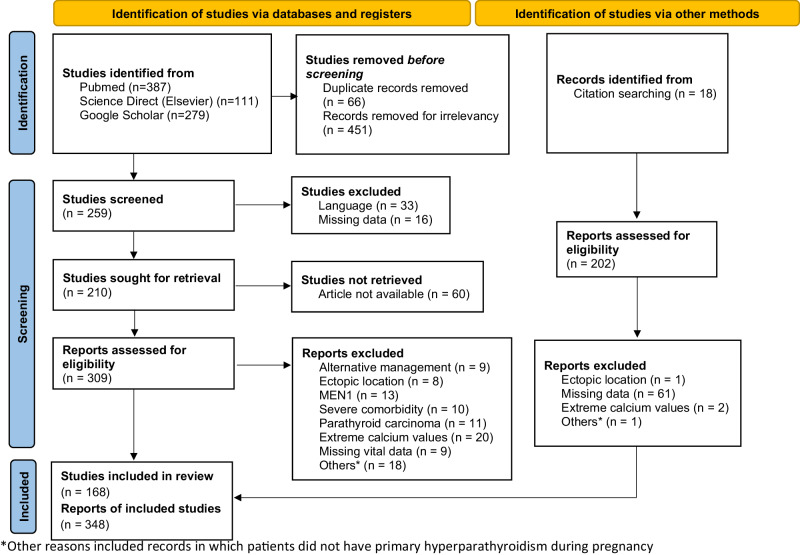
PRISMA flowchart indicating the process for identification and selection of the included studies. *Other reasons included records in which patients did not have primary hyperparathyroidism during pregnancy

The initial search yielded 777 articles, manually filtered to identify 259 relevant articles for screening. Following the screening process for duplications and exclusion criteria, 168 publications were selected, comprising 348 cases for data analysis (as depicted in the PRISMA flow diagram in Fig. 1). Each pregnancy was analyzed as a single case. Maternal, obstetric, and neonatal complications were considered as adverse outcomes that occurred solely during pregnancy or shortly after delivery. Hypercalcemia was defined as total serum calcium exceeding 10.5 mg/dL. Normal range for PTH were defined as 10–65 pg/mL. Calcium values utilized for analysis represented the average of total serum calcium values measured during pregnancy, as ionized calcium was not consistently reported in a significant number of articles. In cases where albumin levels were provided or corrected calcium values were available, the corrected values were utilized for analysis.

### Statistical analysis

Analysis was performed by the Beer-Sheva Faculty of Health Sciences in Israel using Windows GraphPad Prism version 10.2.2. Demographic data is expressed as either raw values or medians and interquartile ranges (IQR) when data did not follow a normal distribution. Statistical significance was assumed as p < 0.05. Differences in complication rates were calculated using the t-test was for quantitative data, and chi-square test for categorical data. Correlation between calcium rates and complication rates was calculated using Pearson’s correlation.

## Results

Out of the 348 cases analyzed, 163 (47%) underwent parathyroidectomy (the surgical group), while 185 (53%) were managed conservatively (the non-surgical group). The mean ages were 31 ± 3 and 31.5 ± 3.5 years, respectively. Average total calcium levels for the surgical group were higher, with a median of 12.3 mg/dL (range:11.5–13.3), compared to 11.1 mg/dL (range: 10.7–11.6) in the non-surgical group. Median PTH levels were 137 pg/mL (range: 94–237) and 123 pg/mL (range:71–224) for the respective groups. 61% of the surgical group underwent surgery during the second trimester, 25% during the third trimester, and 7% during the first trimester. (see Table 1)

**Table 1:** Demographic data of the study population

	Surgical group (n = 163)	Non-surgical group (n = 185)	Both Arms (n = 348)	p-value
Age				
Median (IQR)	31 (28–34)	31.5 (28–34)	31 (28–34)	0.794
Calcium, mg/dL				
Calcium, mg/dLMedian (IQR)	12.3 (11.5–13.1)	11.1 (10.7–11.6)	11.6 (10.8–12.5)	<0.001
PTH, pg/mL				
Median (IQR)	137 (94–237)	123 (71–224)	130 (80–232)	0.89
Phosphorous, mg/dL				
Median (IQR)	2.1 (1.8–2.4)	2.3 (1.9–2.8)	2.1 (1.9–2.5)	0.125
Vitamin D, ng/mL				
Median (IQR)	18 (10.1–27.4)	10.5 (7.7–22.0)	15 (9.1–25.4)	0.6444
Etiology				
Adenoma (n)	85 (88.5%)	40 (93%)	125 (90%)	
Hyperplasia (n)	11 (11.5%)	3 (7%)	14 (10%)	
Week of diagnosis				
Median (IQR)	16 (8–24)	24 (6–30)	17 (8–27)	
Cases diagnosed before pregnancy (n)	17	22	37	
Cases diagnosed retrospectively postpartum (n)	0	20	20	

In the non-surgical group, most patients were treated using IV fluids. Data regarding specific therapeutic agents and their outcomes were missing for most cases; 18 patients received furosemide, an additional 18 received calcitonin and 15 patients received cinacalcet. Positive results to treatment were described in only 17 patients. Thus, statistically significant results regarding the efficacy of different pharmacological regimens could not be achieved.

Maternal, obstetric, and neonatal complication rates comparing the study arms are presented in Table 2 with the following results: Maternal complications occurred in 19.5% of the entire study population. No significant differences in maternal complication rates were observed between the study arms. The overall obstetric complications were not significantly different between study groups as well, however, subcategory analysis revealed higher rates of preeclampsia/eclampsia and preterm labor in the non-surgical group and higher rates of hyperemesis gravidarum in the surgical group. Rates of pregnancy loss were higher in the non-surgical group, with 15.3% compared to 1.3% in the surgical group. From a total of 22 cases of pregnancy loss, 10 cases described 1st-trimester miscarriage, and the rest did not specify further details. Numbers of neonatal complications were significantly higher in the non-surgical group compared to the surgical group. This difference was evident in transient neonatal hypocalcemia (24.4% vs. 2.7%), hypocalcemic tetany (10.7% vs. none), hypocalcemic convulsions (6.9% vs. none), and ICU admissions (9.9% vs. 3.3%), but not neonatal demise.

**Table 2:** Comparison of the maternal, obstetric, and neonatal outcomes between the surgical and non-surgical groups

Maternal complications	Surgical group	Non-surgical group	Both arms	p value
None	79.0%	81.8%	80.5%	NS
Any complication	21.0%	18.2%	19.5%	
Nephrolithiasis	13.4%	10.5%	11.8%	NS
Pancreatitis	4.5%	3.9%	4.1%	NS
Bone pathologies	1.9%	5.0%	3.6%	NS
UTI	3.2%	0.6%	1.8%	NS
ICU admission	1.3%	1.1%	1.2%	NS
Hypercalcemic crisis	0.6%	1.1%	0.9%	NS
Obstetric complications				
None	70.6%	65.9%	68.1%	NS
Any complication	29.4%	34.1%	31.9%	
Preterm labor	9.2%	21.6%	15.8%	<0.005
Preeclampsia / Eclampsia	3.7%	10.3%	7.2%	0.017
Pregnancy loss	1.3%	15.3%	7.8%	<0.005
Hyperemesis gravidarum	11.0%	2.7%	6.6%	<0.005
Gestational hypertension	8.6%	4.9%	6.6%	NS
Emergency delivery	4.3%	6.5%	5.5%	NS
IUGR	2.5%	3.2%	2.9%	NS
Polyhydramnios	1.8%	1.1%	1.4%	NS
GDM	1.2%	0.5%	0.9%	NS
Neonatal complications				
None	90.0%	51.9%	73.3%	<0.001
Any complication	10.0%	49.6%	26.7%	
Transient hypocalcemia	2.7%	24.4%	12.8%	<0.001
Hypocalcemic tetany	0.0%	10.7%	5.0%	<0.001
ICU admission	3.3%	9.9%	6.4%	0.024
Hypocalcemic convulsions	0.0%	6.9%	3.2%	<0.001
Prematurity complications	1.3%	0.8%	1.1%	NS
Neonatal demise	0.0%	1.5%	0.7%	NS

*NS* non-significant, *IUGR* intra-uterine growth restriction, *ICU* intensive care unit, *GDM* gestational diabetes mellitus

**Fig. 2:**
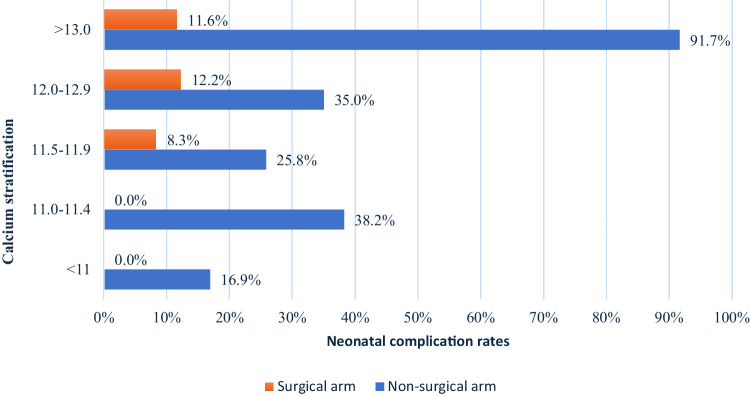
Comparison of neonatal complication rates according to calcium stratification between surgical and non-surgical groups. *p-value for the overall data shown is <0.001

A statistically significant positive correlation was observed between serum calcium values and both maternal and obstetric complication rates (p < 0.05), but not neonatal complications (Table 3). Nevertheless, complication rates in the non-surgical group were significantly higher across all calcium levels compared with the surgical group (with p < 0.001, as illustrated in Fig. 2).

**Table 3:** Maternal, obstetric, and neonatal complications in the entire study population, classified according to mean maternal gestational calcium values

Calcium levels (mg/dL)	Maternal complications		Obstetric complications		Neonatal complications	
	Subjects (n)	Complication rates (%)	Subjects (n)	Complication rates (%)	Subjects (n)	Complication rates (%)
<11	94	10.6%	94	25.5%	52	13.8%
11.0–11.5	55	14.5%	55	23.6%	44	29.5%
11.5–12.0	51	15.7%	55	30.9%	52	19.2%
12.0–12.5	33	27.3%	39	33.3%	36	13.9%
12.5–13.0	30	33.3%	30	43.3%	27	29.6%
>13.0	55	27.3%	55	45.5%	51	31.4%
p-value	0.007		0.002		NS

## Discussion

### Maternal complications

Maternal complications affected 19.5% of the study population in the current study, with no significant difference in complication rates between the surgical and non-surgical groups. Interestingly, maternal complications in the surgical group were not elevated despite the higher mean serum calcium levels. This outcome could potentially be attributed to parathyroidectomy. The variations in calcium levels between the study groups most likely stem from the selection of patients in the higher spectrum of mean serum calcium towards surgical intervention, rather than non-surgical management. Schnatz et al. hypothesized that adverse outcomes in patients selected for surgical intervention could stem from underlying long-term untreated disease rather than from surgery itself [31, 32]. The risk of surgery has been reported to be minimal [28, 33–35], with curative results in 95–98% of cases [9, 14, 36, 37]. In the present study, the operation was curative in 98% of cases, with postoperative complications occurring in 4.9%. Specifically, three patients experienced hungry bone syndrome, three suffered from hypocalcemic tetany, one patient had permanent hypoparathyroidism, and one patient experienced transient vocal cord palsy.

### Obstetric complications

Obstetric complications resulted in significant differences on a few parameters, namely, preterm delivery, preeclampsia, and pregnancy loss, which occurred at higher rates in the non-surgical group. Additionally, hyperemesis gravidarum occurred at higher rates in the surgical group, most likely due to the early diagnosis of their symptomatic disease and subsequent selection for surgical intervention (Table 2). Analysis of the data presented in this article suggests that the increase in pregnancy loss arises from losses that occurred in the first trimester and early second trimester before potential surgical intervention. Consequently, these instances were categorized under the non-surgical group. The pregnancy loss rate for the entire study population was 7.8%, lower than the documented 15% rate for women in the general population aged 30–34 [38, 39]. Overall, the current study findings suggest that parathyroidectomy did not significantly alter the overall rates of obstetric complications when compared to non-surgical management, aligning with the conclusions reported previously by Hirsch et al. [23] and Abood and Vestergaard [22], however, it may potentially be associated to a reduced risk of preterm delivery and preeclampsia.

### Neonatal complications

Neonatal adverse outcomes were significantly more prevalent in the non-surgical group than in the surgical group. The significant difference was evident across all maternal mean calcium values (Fig. 2). These results are supported by previously reported data [30, 40, 41]. Sandler et al. that revealed that even in asymptomatic PHPT, infant complications were less prevalent in the surgical group [41]. The variation in adverse outcomes between the groups primarily consisted of transient postpartum hypocalcemia, hypocalcemic tetany, convulsions, and subsequently, a higher number of ICU admissions. Neonatal hypocalcemia can be severe and prolonged, often necessitating long-term calcium supplementation [42], with median onset of clinical manifestations at the 11th day postpartum [7]. Although a significant correlation was observed between calcium values and maternal and obstetric adverse outcomes, no correlation was seen between neonatal complications and mean maternal gestational calcium values, indicating that neonatal adverse outcomes result from complex interactions beyond mean calcium values alone. Neonatal hypocalcemia is attributed to the suppression of parathyroid glands in utero. After birth, the neonate relies on kidney reabsorption and intestinal absorption, facilitated by an active PTH and calcitriol-dependent mechanism [3]. However, the suppressed parathyroid glands are unable to meet the increased demand, leading to hypocalcemia within the first days to weeks after delivery. During gestation, most calcium is actively transported through the placenta, regulated by PTHrP. 80% of mineral requirements reaches the fetus during the third trimester of pregnancy [7, 43]. Notably, PTH itself does not cross the placenta, and it remains uncertain whether the hormone affects the transfer of calcium through the placenta based on animal models [3]. In this study, most patients in the surgical group underwent parathyroidectomy during the second trimester, indicating their mean gestational calcium during the third trimester remained within normal values. This observation could potentially account for the reduced risk of postpartum neonatal hypocalcemia and its sequelae in the surgical group. However, the limited number of cases specifying post-operative and subsequent gestational serum calcium values made it challenging to draw statistically significant conclusions. Similarly, due to data unavailability, it was impossible to compare the efficacy and outcomes between different non-surgical treatment regimens. Future prospective studies may investigate whether reduced third-trimester calcium values correlate with lower rates of neonatal complications, preferably utilizing ionized calcium, as this marker is more reliable during pregnancy, and compare it with the outcomes of non-surgical management.

## Conclusions

Adverse neonatal outcomes were significantly fewer in the surgical group than in the non-surgical group. This difference was evident across all mean maternal gestational calcium values, suggesting that surgical intervention may yield superior neonatal outcomes, regardless of maternal calcium values. Maternal and overall obstetric complication rates did not significantly differ between the study groups. Additionally, the present study identified a positive correlation between maternal and obstetric outcomes and mean maternal gestational calcium levels, consistent with previously published findings. Given the superior neonatal outcomes alongside non-inferior maternal and obstetric outcomes, the overall data presented in this study suggest that parathyroidectomy is favorable over conservative treatment, even in cases of mild primary hyperparathyroidism.

## Limitations

The primary limitation of this study was the compilation of a patient database from published sources comprising retrospective studies, case series, and case reports. This method introduced a selection bias toward more severe cases, typically reported in the literature. However, this bias is partially mitigated by including 249 cases identified through retrospective reviews. Nevertheless, it cannot be ruled out that asymptomatic patients and those with PHPT within the normocalcemic range may exhibit lower complication rates than those documented in this study.

## Supplementary information

Supplemental Table 1

Supplemental Table 2

## Data Availability

This article has utilized data from widely available, previously published data. All data supporting the findings of this study are available within the paper and its supplementary information which is available in an online repository (10.17632/6vjfskyvzc.2). The Search algorithm is depicted in Table 1, and anonymous case data is in Table 2 of the online repository.
