# Correction: Comparison between surgical and non-surgical management of primary hyperparathyroidism during pregnancy: a systematic review

**DOI:** 10.1007/s12020-024-03987-x

**Published:** 2024-08-19

**Authors:** Shezifi Eli, Shlomo Gozlan Gal, Zaina Adnan

**Affiliations:** 1 Bar-Ilan University, The Azrieli Faculty of Medicine, Safed, Israel; 2 Laniado Hospital, Netanya, Israel; 3 Department of Physiology and Cell Biology, Ben-Gurion University of the Negev, Beer-Sheva, Israel; 4 Division of Endocrinology and Metabolism, Clalit Medical Health Care Services, Haifa and Western Galilee District, Zvulon Medical Center, Haifa, Israel

Correction to: *Endocrine*

10.1007/s12020-024-03930-0, published online 25 June 2024

In the original version of this article, in the “Abstract” section “Conclusion” heading the sentence “significantly lower neonatal adverse outcomes in the non-surgical group compared to the surgical group” should read as “significantly lower neonatal adverse outcomes in the surgical group compared to the non-surgical group”.

The original article has been corrected.

