# Supplementary material for: Comparison between surgical and non-surgical management of primary hyperparathyroidism during pregnancy: a systematic review
Source: Endocrine. 2024 Jun 25;86(1):101–8. doi: 10.1007/s12020-024-03930-0 (PMC11445326; doi:10.1007/s12020-024-03930-0)
Supplement: Supplementary file 1 — Supplemental Table 1 [file 12020_2024_3930_MOESM1_ESM.docx]

**Supplemental Table 1** Search Algorithm.

| Databases | Search algorithm | Filters |
| --- | --- | --- |
| Science Direct (Elsevier) | (Pregnancy OR gestation) AND (hyperparathyroidism OR parathyroidectomy OR PHPT) | 1980-2023 |
| PubMed/MEDLINE | (Pregnancy OR gestation) AND (hyperparathyroidism OR parathyroidectomy OR PHPT) | Human species, case reports, reviews, systematic review, meta-analysis, 1980-2023 |
| Google Scholar | Pregnancy AND hyperparathyroidism | In: title |
